# Food Security and Nutrition Outcomes of Farmer Field Schools in Eastern Democratic Republic of the Congo

**DOI:** 10.9745/GHSP-D-17-00203

**Published:** 2017-12-28

**Authors:** Shannon Doocy, Sarah Cohen, Jillian Emerson, Joseph Menakuntuala, the Jenga Jamaa II Study Team, Jozimo Santos Rocha

**Affiliations:** a Johns Hopkins Bloomberg School of Public Health, Baltimore, MD, USA.; b Adventist Development and Relief Organization, Silver Spring, MD, USA.; c Includes Kimberly Amundson Mansen, Laura Caulfield, Elizabeth Colantouni, Rolf Klemm, and Johnathan Strong from the Johns Hopkins Bloomberg School of Public Health, Baltimore, MD, USA, and Laura Brye, Sonya Funna, Jean-Pierre Nzanzu, and Espoir Musa from the Adventist Development and Relief Organization, Silver Spring, MD, USA.

## Abstract

**Background:**

Food and nutrition security in eastern Democratic Republic of the Congo are threatened by political instability and chronic poverty. The Jenga Jamaa II project, implemented between 2011 and 2016 in South Kivu Province, aimed to improve household food security and child nutritional status using various intervention strategies, including farmer field school (FFS) programs.

**Objective:**

To characterize the changes in agricultural production techniques, household food security, and child nutritional status associated with participation in FFS programs.

**Methods:**

We used a community-matched design to select FFS intervention and control households from 3 health zones in which the project was operating. Data on food security (Household Dietary Diversity Score [HDDS] and Household Food Insecurity Access Scale [HFIAS]) and child anthropometry were collected semiannually for 3.5 years in both groups. Additional data on agricultural practices were collected annually in the FFS group only. Focus groups with FFS staff and beneficiaries were conducted in the final project year. Statistical analyses included basic descriptive statistics such as paired *t* tests and analysis of covariance; regression models using a bootstrap were applied to generate *P* values and confidence intervals while accounting for differences between groups.

**Results:**

The study enrolled 388 FFS beneficiaries and their households in the intervention group and 324 non-FFS households in the control group. FFS participants reported increasing the number of different agricultural techniques they used by an average of 2.7 techniques over the project period, from 5.1 in 2013 to 7.9 in 2016 (*P*<.001). The mean HDDS and HFIAS improved more in the FFS group than in the control group (mean difference between intervention and control for HDDS was 0.9 points and for HFIAS was −4.6 points; *P*<.001). However, the prevalence of child stunting (60.2% intervention vs. 58.8% control) and underweight (22.3% intervention vs. 29.8% control) were similar in both groups at endline (*P*>.05).

**Conclusion:**

Although FFS participants diversified their agricultural production strategies and experienced improvements in household food security, there was not a positive impact on child nutritional status. In this food-insecure context, improvements in agricultural production alone are unlikely to significantly change child nutritional status—a health outcome with a complex, multilevel causal chain.

## Abstract (precis)

A farmer field school program in food-insecure areas had positive impacts on household food security but not child nutritional status. Similar agricultural interventions may benefit food security, but the more difficult-to-achieve improvements in child nutrition status may require more focused and integrated programming approaches.

## INTRODUCTION

The eastern provinces of the Democratic Republic of the Congo (DRC) have been in a protracted state of emergency. Between 1998 and 2007, there were an estimated 5.4 million excess deaths, many of which occurred after the war officially concluded in 2002; most mortality was the result of nonviolent causes including malnutrition, diarrhea, and maternal complications.^1^ The conflict has disrupted agricultural production and decreased harvests.^2^ South Kivu, a region where insecurity and violence endure today,^3^ is one of the most food- and nutrition-insecure provinces in the country.^4^ Stunting affects more than half (53%) of children under 5 in South Kivu—the highest prevalence nationwide. Underweight prevalence, at 26%, is similarly high relative to other provinces, and almost two-thirds of households are moderately or severely food-insecure.^5^^,^^6^

The relationship between agriculture and nutrition is complex. While it may seem obvious that agriculture influences nutrition, numerous reviews have determined that the data linking agricultural interventions to nutritional impacts are often either inconclusive or incomplete.^7^^–^^12^ These reviews covered a diverse range of agricultural interventions (e.g., training and/or material inputs for home gardens, household livestock production, or biofortification), which in some instances successfully increased agricultural production.

These reviews did not focus on farmer field school (FFS) programs, which are increasingly widespread development approaches to strengthen farmers' capacity to adopt ecologically friendly technologies and crop management practices, and ultimately to increase crop yields. FFS programs, which were first developed in Indonesia in 1989, are now used globally with adaptations in context and approach based on the local environment (for example, in sub-Saharan Africa, the topics covered have been expanded to include nutrition and malaria).^13^

Farmer field schools are increasingly common approaches to improving both ecological practices and crop yields.

FFS programs employ a participatory approach in which groups of farmers, steered by facilitators, engage in practical investigations, observations, and synthesis.^14^ Findings on the benefits of FFS programs are mixed: some studies have shown that FFS programs have potential to change participants' agricultural practices^15^ and increase revenue and productivity,^16^ while other studies have suggested that FFS participation alone does not necessarily increase crop yields in the long term, but rather that additional interventions are required in combination with FFS for such gains.^17^^,^^18^ Such mixed results may relate to study methodology; case studies generally show positive impacts possibly reflective of short-term effects, while relatively little improvement may be observed in longitudinal studies measuring medium-term impacts.^18^

Although questions have been raised about the cost-effectiveness and sustainability of FFS programs, there is support for the FFS approach and its benefits for participants.^13^ Few studies to date have examined the food security and nutritional impacts of FFS programs. There is a complex relationship between agriculture, food security, and child nutrition, and this article examines the changes in agricultural production practices, household food security, and child nutritional status that are associated with participation in FFS programs. Our goal is to contribute to the wider body of literature concerning linkages between agriculture and health.

## METHODS

This article characterizes outcomes of an FFS intervention that was one component of the Jenga Jamaa II project, a development food assistance program funded by the U.S. Agency for International Development (USAID) Office of Food for Peace. Jenga Jamaa II sought to address household food insecurity and child undernutrition. It was implemented by Adventist Development and Relief Agency (ADRA) in Fizi and Uvira territories of South Kivu Province between 2011 and 2016 (Figure) and reached more than 258,000 beneficiaries with the following objectives:

1. Increasing incomes among farming households through FFS and farmer-to-farmer training interventions
2. Improving the health and nutritional status of children under 5 years of age through the Preventing Malnutrition in Children under 2 Approach (PM2A)
3. Empowering women via women's empowerment groups

Jenga Jamaa II was a 5-year development food assistance program that sought to address food insecurity and child undernutrition.

The research reported here derives from a subset of the data from the parent study of Jenga Jamaa II outcomes. Additional findings, including a comparison of all interventions, are presented elsewhere, along with more detailed information about overall study methods and statistical analyses.^19^^,^^20^ Here we summarize the methods relevant to the current research, as well as information specific to the FFS analysis.

**FIGURE:**
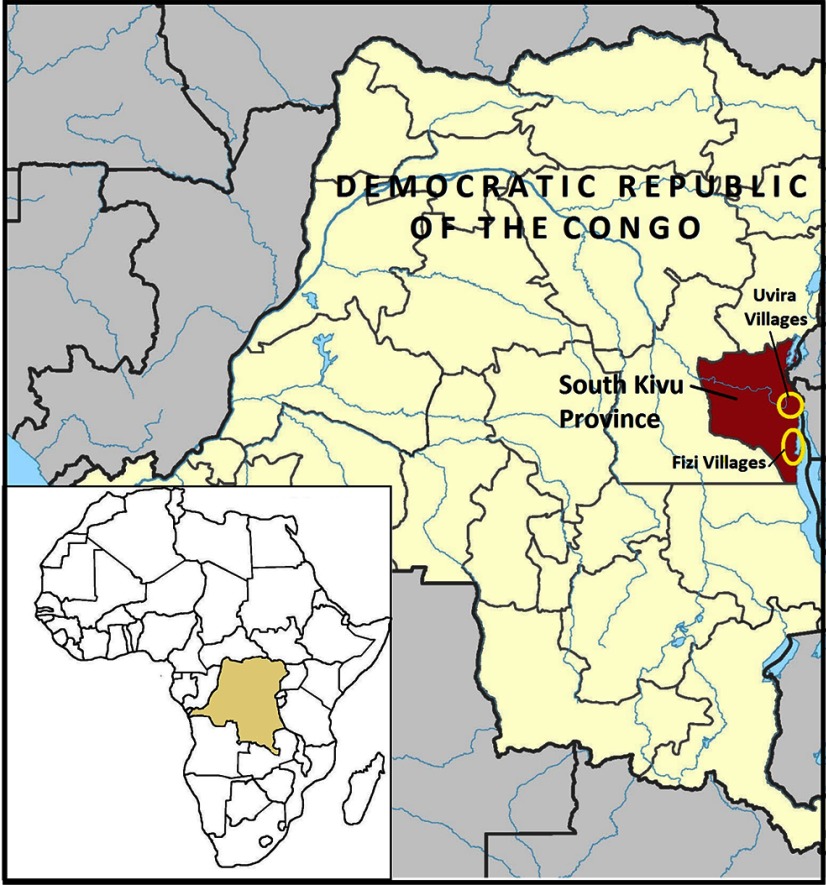
Map of the Jenga Jamaa II Project and Study Area Adapted from User:Profoss. File:Democratic Republic of the Congo (26 provinces)- Sud-Kivu.svg. Wikimedia Commons. February 16, 2016. https://commons.wikimedia.org/wiki/File:Democratic_Republic_of_the_Congo_(26_provinces)_-_Sud-Kivu.svg. Accessed November 28, 2017.

### Intervention

The FFS intervention provided farmers with experience-based education on farming practices and postharvest handling as well as business and natural resource management skills. Each FFS group received semimonthly trainings from ADRA field agents for 2 years. Each FFS group had a community demonstration plot, and group members also received starter packages of seeds and tools for use on individual farms. The FFS programs focused on a variety of common crops in the region, including cassava, maize, rice, beans, banana, and peanuts. The first year of training focused on knowledge of production systems and technologies; adoption of techniques and technologies and behavior change were the focus in the second year. Content was designed to be crop-specific and seasonally appropriate. After completing the FFS intervention, many beneficiaries transitioned to farmer business associations, which were intended to improve access to credit and marketing opportunities.^19^

The Jenga Jamaa II farmer field schools provided education on farming practices, postharvest handling, and business and natural resource management skills.

### Sample Size

For this article, we analyzed 2 of the 5 comparison groups recruited for the parent study of Jenga Jamaa II: the FFS intervention group (388 participants) and the control group (324 participants). The primary outcome measure was reduction in household food insecurity, and we conducted calculations for varying levels of reduction, assuming 80% power and a significance level of .05. With a minimum sample size of 325 households per group (or 1,625 households in total for the parent study), the study was powered to detect a 10% or greater reduction in prevalence of food insecurity indicators within each comparison group, as compared to baseline levels.^19^

### Study Design and Data Collection

The Jenga Jamaa II parent study used a quasi-experimental matched design in which communities planned to receive 1 intervention (versus multiple interventions) selected for participation so that the effect of individual interventions could be assessed. The 4 Jenga Jamaa II interventions were women's empowerment groups, PM2A, FFS, and farmer-to-farmer training; a fifth comparison group was recruited as a control group. Participating communities within each territory (Fizi and Uvira) were matched by livelihood zone (mountains, plains, or lakeside) and proximity into sets of villages with each type of intervention. The final sample had 13 sets of 3 villages; within each set of villages, one village received agricultural interventions, the second received PM2A, and the third received women's empowerment groups. In each set of villages, intervention groups were formed (i.e., 1 intervention per village) and all beneficiaries in the group were enrolled in the study. In agricultural intervention villages, the entire FFS group of approximately 30 beneficiaries was enrolled in the study. Controls were selected from women's empowerment group villages, where each beneficiary was matched with a female neighbor not participating in Jenga Jamaa II interventions, and that woman's household was enrolled as a control.

The Jenga Jamaa II project enrolled study participants between August and October 2012, following identification of beneficiaries for each intervention. A total of 1,820 beneficiaries and their households were enrolled; this included 1,385 child household members born between July 2010 and December 2012 (this age group was identified specifically for the PM2A intervention). All children in enrolled households born during the eligibility period were included in the anthropometric assessments.

Study households were followed for 3.5 years, from enrollment in the fall of 2012 (baseline) through February or March 2016 (endline), regardless of whether the Jenga Jamaa II beneficiary graduated or dropped out of the intervention. Data were collected in 8 semiannual surveys (August/September and February/March) to account for seasonal variations in food security.^20^ Both data collection periods were at the beginning of local rainy seasons.^21^

The survey questionnaire focused on measures of food security, household economy, dietary intake, and nutritional status. The study team also collected annual data from the FFS group on agricultural practices related to production, adoption of farming practices, postharvest storage methods, and 18 agricultural technologies (mulching, crop rotation, row planting, weeding, contour lines, hoeing, organic fertilizer, intercropping, organic pesticide, mounding, improved seeds, resistant cassava varieties, resistant banana suckers, animal traction, sprayers, tractors, other techniques, other technologies).

The questionnaire was developed using validated measures such as those from Demographic and Health Surveys, food security assessments, and Food for Peace program indicators.^19^ Food security indicators included the Household Dietary Diversity Score (HDDS) and the Household Food Insecurity Access Scale (HFIAS),^22^^–^^24^ both of which are validated and widely used, including as Food for Peace program indicators. HDDS is a proxy measure of household food access that assesses household dietary quality based on reported consumption of 12 food groups in the preceding 24 hours; households consuming 5 or more food groups are classified as achieving target dietary diversity.^23^ HFIAS measures household food insecurity over the preceding month using a 9-item questionnaire that measures key domains of food access; responses are summed to create a total score between 0 (most food secure) and 27 (most food insecure), which can also be interpreted categorically.^24^ The questionnaire was developed in English and translated to Swahili, the predominant local language; it was finalized after Jenga Jamaa II pilot testing and translation review.^19^

Semiannual surveys assessed household food security and dietary diversity.

The primary measures for child growth were stunting, or low height-for-age, and underweight, or low weight-for-age. Anthropometric data (weight and height) were collected at each semiannual survey for all children in enrolled households born between July 2010 and December 2011. Weight was measured using Tanita Mommy and Baby Infant Scales, Model 1582 (Arlington Heights, IL, USA), and Shorr Productions height boards (Olney, MD, USA); recumbent length was measured for children 6 to 23 months of age and height for children older than 24 months.^20^

The study began collecting data with paper questionnaires, then transitioned to electronic data collection, using the Magpi platform (Datadyne, LLC), approximately halfway through the study. Due to high levels of illiteracy, oral consent was obtained at enrollment and at each subsequent survey; participants were reminded that participation was voluntary and that declining to participate in the study would not affect benefits received from participating in Jenga Jamaa II. Study participants received a small incentive, most often soap, worth approximately US$1, for participation in each survey.^20^

Qualitative research included 7 focus groups of 6 to 8 FFS beneficiaries; 2 group interviews with ADRA field agents; and 7 individual key informant interviews with community leaders. ADRA management and technical staff also participated in interviews and responded to queries related to program delivery. Qualitative data collection occurred at the end of the project period and focused on (1) overall perceptions of problems currently faced by communities, (2) if and how the FFS intervention helped to mitigate these difficulties, and (3) identifying which program elements were most useful and most challenging. Focus group and key informant interview results were analyzed using qualitative description and content analysis.

Focus groups and interviews at the end of the project solicited feedback on if and how the farmer field schools helped address identified community problems.

### Statistical Analysis

Data analysis was performed using Stata 13 (StataCorp, 2013). Exploratory analysis included calculating unadjusted means and prevalence of binary indicators for each survey and identifying outliers; assessing patterns of missing data and dropouts across study groups and assessing differences in outcomes between those who had dropped out or been absent for the previous survey and those who had not; and assessing correlation over time using autocorrelation matrices for continuous outcomes and lorelograms for binary outcomes.^25^

With the exception of imputation procedures for the child anthropometric data, our analysis did not consider interim measures of each indicator because after exploratory analysis, it became clear that inclusion of interim data points did not change results and conclusions, and thus eliminating analysis of interim measures would facilitate the interpretation of findings. The strengths of the analysis are (1) its use of propensity scores to account for differences between groups, (2) its ability to account for baseline differences in the outcome indicators between groups, and (3) its controls for differences in territory and livelihood zones (mountains, plains, or lakeside).

The results presented include only the 82% of study participants who were present for both baseline and endline surveys (both of which were conducted in the February/March time period). The village of Kibirizi, which included 1 FFS group, was not included in the final endline survey (and thus was excluded from the final evaluation) due to security concerns. Despite our inability to access Kibirizi for the final survey, participant follow-up was considered high given the context and the 3.5-year data collection period that was necessary for adequate assessment of medium-term changes in food security indicators.^19^

To estimate differences in outcomes between groups over time, we used analysis of covariance (ANCOVA) to estimate mean change in the outcome variable. We compared the last follow-up to baseline for each treatment group separately; the outcome at endline for the intervention group was then compared to the endline outcome for the control group. ANCOVA allows precise estimates by accounting for chance imbalance across intervention groups in baseline variables that are prognostic for the outcome of interest (e.g., stratification variables and the baseline outcome). We used a linear model for the outcome at the last follow-up, with main terms for the intervention group (4 dummy variables), the baseline outcome, and 2 stratification variables (territory and livelihood zone). Maternal age and education were also included in models for child diet and nutritional outcomes. For binary outcomes, prevalence at the last follow-up was estimated for each intervention group; the treatment effect was defined as the difference in prevalence found by comparing each intervention group to the control. The analysis included adjustment for the stratification variables, baseline outcome in the case of child outcomes, and maternal characteristics. To estimate the treatment effects, an outcome regression estimator referred to as the doubly robust weighted least squares estimator was used, which is analogous to the ANCOVA approach but applies to non-continuous outcomes.^26^^,^^27^ Standard errors, confidence intervals (CIs), and *P* values were generated using a bootstrap.

Anthropometric *z* scores for children 6 to 59 months of age were calculated using the 2006 World Health Organization (WHO) child growth standards with the user-written Stata program zscore06.^28^ Anthropometric *z* scores for children over 5 years of age were calculated using the 2007 WHO reference for children 5 to 19 years, using the Stata program zanthro.^29^ Children with a height-for-age *z* score (HAZ) less than −2 were classified as stunted, and those with a HAZ less than −3 as severely stunted; similarly, children with a weight-for-age *z* score (WAZ) less than −2 were classified as underweight and those with a WAZ less than −3 as severely underweight. We used a multiple imputation approach for anthropometric outcomes, where missing values were replaced by values sampled from a distribution defined by the fit of a linear regression model at a given follow-up as a function of previous outcomes, as well as of child age and sex.

The methods described here were applied to each survey data set and then averaged using Rubin's method to obtain final estimates.^30^ Propensity scores were used to account for the non-randomized design. Propensity score weights were defined using beneficiary age, sex and education (for the control group maternal age and education were used in lieu of beneficiary characteristics); household landownership and number of income sources; and number of children under 2 years old in the household. Models for child outcomes accounted for within-household clustering. Children who died were excluded from the analysis, and missing values for maternal age and education were assigned the mean and mode of those variables, respectively, so they could be included in the analysis. The model coefficient for the FFS group represents the estimated difference compared to the control group.^20^

### Ethical Approvals

Approval to conduct the parent study was obtained from local authorities in the relevant administrative areas of South Kivu and from the Institutional Review Board of the Johns Hopkins Bloomberg School of Public Health.

## RESULTS

### Demographic Characteristics

This study enrolled 388 FFS beneficiaries and their households in the intervention group and 324 non-FFS adults and their households in the control group (Table 1). For the control group, we enrolled the primary caretaker of children, and 100% were women. For the FFS group, we enrolled the FFS beneficiary and his or her household, and 69% of those enrolled were women (*P*<.001). The FFS and control groups were similar with respect to household size (median=6), but the intervention group households had significantly fewer young children (*P*<.001 for both children under 2 years and children between 2 and 4 years old). The groups were similar with respect to land ownership (69% owned land), and more than 95% of households in each group reported having farmers, though this proportion was significantly higher in the FFS group (*P*=.007). FFS beneficiaries were significantly older than those enrolled as control group participants (mean age 38 years vs. 31 years, *P*<.001). In both groups, maternal educational attainment was low overall, with over 90% of FFS group mothers and 74% of control group mothers having not completed any formal schooling (*P*=.002).

**TABLE 1.** Baseline Demographic Characteristics for Intervention and Control Groups, 2012

	Intervention Group: FFS Beneficiaries (n=388)	Control Group: Non-FFS Participants (n=324)	*P* Value^a^
**Respondent Characteristics**^b^			
Sex, % Female	69.4	100	**<.001**
Age, years			
Median	35	28	–
Mean (SD)	37.9 (13.4)	31.1 (10.2)	**<.001**
Highest level of education, %			.07
None	72.1	75.0	
Primary	25.0	25.0	
Secondary	2.9	0.0	
**Household Characteristics**			
Household size			
Median	6	6	–
Mean (SD)	6.2 (2.4)	6.3 (2.4)	.58
Maternal highest level of education, %			
None	90.1	74.5	
Primary	9.9	24.5	**.002**
Secondary	0.0	1.0	
Maternal age			
Median	29	28	–
Mean (SD)	32.8 (11.3)	31.1 (10.2)	.15
Number of children ages 2–4 years			
Median	1	2	–
Mean (SD)	1.5 (1.1)	1.8 (1.1)	**<.001**
Number of children ages <2 years			
Median	0	1	–
Mean (SD)	0.5 (0.5)	0.7 (0.5)	**<.001**
Households with farmer, %	98.7	95.4	**.007**
Households owning farmland, %	69.4	68.6	.85

Abbreviations: FFS, farmer field school; SD, standard deviation.

a *P* values in boldface indicate differences significant at the *P*<.05 level. *P* values were generated from Pearson's chi-square test for binary and categorical variables, and *F* test for means (analysis of covariance, or ANCOVA) for continuous variables.

b FFS respondents were the primary beneficiaries of the FFS intervention, whereas respondents in the control group were most often mothers of children in the household.

### Use of Agricultural Techniques Among FFS Beneficiaries

Over the 4-year intervention period (2012–2016), the number of agricultural techniques and technologies that the FFS beneficiaries used increased from an average of 5.1 reported in 2013 to 7.9 in 2016 (*P*<.001) (Table 2). Of the 18 techniques and technologies assessed, 6 techniques saw both statistically significant increases in use and were used by more than 20% of the FFS households at the end of the project period in 2016. Weeding (96.2%), hoeing (95.9%), and row planting (92.7%) were the most commonly used techniques. Crop rotation, mulching, and row planting had the highest adoption rates, with increases of 58.8%, 48.9%, and 40.4%, respectively, of households adopting the techniques following FFS participation. Statistically significant increases in use of sprayers, organic pesticide, organic fertilizer, tractors, and animal traction were also observed, but adoption was below 20% at the end of the project. No significant changes were observed in use of improved banana suckers, contour lines, hoeing, intercropping, and other techniques. Use of resistant cassava varieties saw a significant decrease (−12.4%, *P*=.02), although adoption remained high at endline (59%); other technologies also decreased significantly (*P*<.001), with adoption under 1% at endline.

**TABLE 2.** Percentage of Intervention Households That Used Agricultural Techniques and Business Development Strategies in the Most Recent Growing Season,^a^ 2013–2016

	2013 (n=370)		2014 (n=350)		2015 (n=388)		2016 (n=317)		Change (2013–2016)	
	Point^b^	95% CI	Point	95% CI	Point	95% CI	Point	95% CI	Point	*P* Value^c^
**Agricultural techniques**^d^										
All techniques (mean)	5.1	(5.0 to 5.4)	6.5	(6.3 to 6.7)	7.6	(7.3 to 7.8)	7.9	(7.7 to 8.2)	2.7	**<.001**
Mulching	28.9%	(24.2 to 34.1)	46.7%	(41.3 to 52.1)	71.5%	(66.7 to 76.0)	77.8%	(72.9 to 82.3)	48.9%	**.001**
Crop rotation	18.1%	(14.2 to 22.6)	32.8%	(27.8 to 38.0)	59.4%	(54.2 to 64.4)	76.9%	(71.9 to 81.4)	58.8%	**.001**
Row planting	52.3%	(46.9 to 57.7)	63.5%	(58.2 to 68.6)	84.1%	(80.0 to 87.6)	92.7%	(89.3 to 95.3)	40.4%	**.001**
Weeding	82.7%	(78.3 to 86.6)	93.0%	(89.8 to 95.0)	97.6%	(95.5 to 98.9)	96.2%	(93.4 to 98.0)	13.5%	**.001**
Contour lines	80.1%	(75.5 to 84.2)	76.5%	(71.7 to 80.9)	72.1%	(67.3 to 76.6)	79.1%	(74.2 to 83.4)	−1.0%	.68
Hoeing	93.3%	(90.1 to 95.7)	97.4%	(95.1 to 98.8)	97.4%	(95.2 to 98.7)	95.9%	(93.1 to 97.8)	2.6%	.38
Intercropping	41.9%	(36.6 to 47.4)	50.4%	(45.0 to 55.8)	49.1%	(43.9 to 54.2)	42.7%	(37.2 to 48.4)	0.8%	.80
Mounding	18.2%	(14.2 to 22.7)	22.0%	(17.8 to 26.8)	30.1%	(25.5 to 35.1)	30.7%	(25.7 to 36.1)	12.5%	**.006**
Improved seeds	41.1%	(35.9 to 46.6)	73.6%	(68.6 to 78.2)	81.0%	(76.7 to 84.9)	75.0%	(69.8 to 79.7)	33.9%	**<.001**
Resistant cassava varieties	71.6%	(66.4 to 76.3)	75.4%	(70.5 to 79.8)	74.1%	(69.4 to 78.5)	59.2%	(53.5 to 64.6)	−12.4%	**.02**
**Marketing strategies**^d^										
Individual	64.0%	(58.4 to 68.9)	71.3%	(66.2 to 76.0)	63.8%	(58.6 to 68.9)	58.8%	(53.2 to 64.3)	−5.2%	.35
Agriculture collection center	0.3%	(0.0 to 1.6)	0.0%	(0.0 to 1.1)	5.5%	(3.4 to 8.4)	30.1%	(25.1 to 35.4)	29.8%	**<.001**
Joint negotiation at FFS level	0.8%	(0.1 to 2.5)	1.4%	(0.5 to 3.3)	22.1%	(17.9 to 26.7)	69.6%	(64.2 to 74.6)	68.8%	**<.001**
Joint negotiation at FBA level	0.3%	(0.0 to 1.6)	0.6%	(0.1 to 2.1)	15.9%	(12.3 to 20.1)	56.6%	(51.0 to 62.1)	56.3%	**<.001**
**Financial services**^e^										
Informal credit	22.6%	(18.3 to 27.4)	4.3%	(2.5 to 7.1)	7.4%	(4.9 to 10.6)	14.2%	(10.6 to 18.6)	−8.4%	**.006**
Savings	7.2%	(4.7 to 10.5)	22.3%	(18.0 to 27.1)	36.7%	(31.8 to 41.9)	50.3%	(44.7 to 56.0)	43.1%	**<.001**

Abbreviations: CI, confidence interval; FBA, farmer business association; FFS, farmer field school.

a “Most recent growing season” refers to the season preceding interviews conducted in February/March of indicated year.

b “Point” refers to point estimate (% or mean) in each column.

c *P* values in bold text indicate differences significant at the *P* < .05 level.

d Results for agricultural techniques with less than 20% adoption at endline (e.g., organic pesticide, organic fertilizer, virus-resistant banana suckers, tractors, animal traction for tillage, sprayers, other techniques, and other technology) are not presented in the table.

e Results for marketing strategies and financial services with less than 10% adoption at endline (e.g., formal credit and insurance) are not presented in the table.

Farmers who attended farmer field schools increased the number of different agricultural techniques they used.

### Use of Marketing and Financial Services Among FFS Beneficiaries

Before FFS participation, the most commonly used marketing strategy was individual crop sales, reported by 64% of households; other marketing strategies were used by less than 1% of households (Table 2). We found high levels of adoption of the various marketing strategies following the FFS intervention, with statistically significant increases in the proportion of households reporting use of joint negotiation at the FFS (68.8%) and farmer business association levels (56.3%), as well as in sales through agricultural collection centers (29.8%; *P*<.001 for all comparisons) (Table 2). Use of financial services also changed over the course of the intervention. Before FFS, informal credit was the most common financial service, used by 22.6% of households, whereas by the end of the project period, use of informal credit decreased by 8.4% (*P*=.006) and use of savings increased by 43.1% (*P*<.001). Both use of formal credit and use of insurance increased during the intervention period, but these increases were not significant and rates of adoption by FFS households were below 10% at the end of the project, due largely to poor access to these types of services in study areas.

After participating in a farmer field school, farmers adopted several new marketing techniques.

### Household Food Security Outcomes

At enrollment, mean HDDS among the FFS group was 3.4; this increased to 5.6 at the end of the project (mean change=2.1; 95% CI, 1.9 to 2.4; *P*<.001) (Table 3). In comparison, a smaller increase, from 3.4 to 4.8, was observed among the control group (mean change=1.4; 95% CI, 1.0 to 1.7; *P*<.001). In the adjusted analysis at endline, the mean difference between the 2 groups was 0.9 points (95% CI, 0.5 to 1.3; *P*<.001). Similarly, the difference in the adjusted proportion of FFS and control households achieving target dietary diversity at endline was 21.7% (95% CI, 12.3 to 31.1; *P*<.001). HFIAS scores decreased by 8.6 points (95% CI, −9.4 to −7.9; *P*<.001) in the FFS group and 4.7 points in the control group (95% CI, −5.7 to −3.7; *P*<.001) over the project period. After adjustment, the difference in mean HFIAS change between the 2 groups was −4.6 points (95% CI, −5.0 to −4.2; *P*<.001). At end of the project period, the proportion of households that improved an HFIAS category was 22.9% higher in the FFS group than in the control group (95% CI, 12.7 to 33.1; *P*<.001).

**TABLE 3.** Differences in Household Food Security Outcomes Between the Intervention and Control Groups

	Intervention Group: FFS Beneficiaries (n=317)	Control Group: Non-FFS Participants (n=254)	Difference Between Groups	*P* Value
**Household Dietary Diversity Score**^a^				
Baseline, mean (SD)	3.4 (1.4)	3.4 (1.5)	–	–
Endline, mean (SD)	5.6 (2.1)	4.8 (2.1)	–	–
Change over time,^b^ adjusted^c^ mean (CI)	2.1 (1.9 to 2.4)	1.4 (1.0 to 1.7)	0.9 (0.5 to 1.3)	<.001
Achieved target at endline,^c^ % (CI)	69.7 (63.6 to 75.9)	48.0 (40.6 to 55.3)	21.7 (12.3 to 31.1)	<.001
**Household Food Insecurity Access Scale**				
Baseline, mean (SD)	14.4 (4.6)	14.8 (5.3)	–	–
Endline, mean (SD)	5.7 (5.1)	10.1 (6.1)	–	–
Change over time,^b^ adjusted^c^ mean (CI)	−8.6 (−9.4 to −7.9)	−4.7 (−5.7 to −3.7)	−4.6 (−5.0 to −4.2)	<.001
Improved a category^c^ (baseline to endline), % (CI)	55.3 (48.8 to 61.9)	32.4 (24.6 to 40.3)	22.9 (12.7 to 33.1)	<.001

Abbreviations: CI, confidence interval; FFS, farmer field school; SD, standard deviation.

a Each point corresponds to a food group.

b Paired *t* test.

c Adjusted for baseline Household Dietary Diversity Score, territory, and agro-ecological zone.

c Adjusted for baseline score on the Household Food Insecurity Access Scale, territory, and agro-ecological zone.

### Child Nutrition Outcomes

At the end of the project, the FFS group had an adjusted stunting prevalence of 60.2% as compared to 58.8% in the control group (Table 4); the 1.4% difference in stunting prevalence between groups was not statistically significant (*P*=.81). Similarly, the FFS group had an adjusted underweight prevalence of 22.3% as compared to 29.8% in the control group, and the 7.6% difference in underweight prevalence between groups was also not statistically significant (*P*=.13).

**TABLE 4.** Differences in Child Nutrition Outcomes at Endline Between the Intervention and Control Groups

	Intervention Group: Children of FFS Beneficiaries (n=265)	Control Group: Children of Non-FFS Participants (n=206)	Difference Between Groups	*P* Value
Adjusted endline stunting prevalence,^a^ % (CI)	60.2 (50.8 to 69.6)	58.8 (50.1 to 67.5)	1.4 (−10.7 to 13.6)	.81
Adjusted endline underweight prevalence,^b^ % (CI)	22.3 (14.8 to 29.8)	29.8 (22.0 to 37.7)	−7.6 (−17.7 to 2.5)	.13

Abbreviations: CI, confidence interval; FFS, farmer field school; SD, standard deviation.

a Adjusted for baseline stunting status, territory, agro-ecological zone, maternal age, and maternal education; children with a height-for-age *z* score less than −2 SD using the 2006 WHO child growth standards (for children ages 6–59 months) and the 2007 WHO reference (for children over 5 years) were classified as stunted.

b Adjusted for baseline underweight status, territory, agro-ecological zone, maternal age, and maternal education; children with a weight-for-age *z* score less than −2 SD using the 2006 WHO child growth standards (for children ages 6–59 months) and the 2007 WHO reference (for children over 5 years) were classified as underweight.

### Beneficiary Perceptions of FFS Programming

Participants described the FFS programs as leading to many benefits. First, they noted that the improved agricultural techniques they learned were helpful, particularly in poor growing seasons. In addition, beneficiaries explained that working in a group improved their leadership skills and ability to work cooperatively. The FBAs that some FFS programs formed were perceived to improve business skills (e.g., assessing markets, setting fair prices, and negotiating jointly via group contracts).

Participants described the farmer field schools as leading to many benefits, including improved agricultural, leadership, and business skills.

Although beneficiaries had generally positive impressions of participation in the FFS, the FFS program was not without challenges. Crop diseases, particularly the emergence of cassava brown streak disease and the widespread prevalence of cassava mosaic disease and banana wilt, posed problems and diminished the participants' harvests. Delayed arrival of seeds was another contributor to weakened production. Participants also referenced obsolete and inefficient tools as barriers to improved income, and proposed mechanization and use of improved technologies (e.g., tractors and motorized mills) as solutions.

### Staff Perceptions of FFS Programming

The challenges described by FFS program staff differed from those recognized by beneficiaries. Staff identified the lack of coordination between organizations in the region as a principal challenge, where the types and amounts of agricultural inputs and incentives provided to beneficiaries varied, leading to disappointment among participants. Staff agreed with participants that late seed arrival posed a significant challenge. In addition, some communities were remote and inaccessible, making it hard for beneficiaries to move their goods to markets. Even with these difficulties, field agents and staff felt that the FFS interventions offered benefits to the participants; for example, by the end of the program, staff indicated that participants were more likely to sell crops to improve their household's dietary diversity, demonstrating a change in mind-set. Furthermore, marketing activities contributed to sales at more stable prices.

## DISCUSSION

Over the course of the 4-year implementation period (from enrollment in 2012 through endline data collection in 2016), participants in the FFS intervention increased use of improved agricultural techniques, diversified their business strategies, and experienced improvements in household food security. However, these gains did not translate into improvements in child nutritional status. These findings highlight the complex relationship between agriculture, food security, and nutrition and the difficulties of achieving sustained changes in health status in low-resource settings. While changes in the yields or income of participating farmers are not presented here, there was a statistically significant increase over time in the number of improved agricultural techniques used, and farmers noted in focus group discussions that these techniques helped improve production; the focus groups also indicated that the diversified marketing and sales strategies were beneficial. Thus, our findings appear to support other reports of the agriculturally beneficial effects of FFS programs.^15^^,^^16^

While many FFS programs aim to improve food security,^31^ there have been relatively few studies that have assessed whether FFS participation impacts household food security. One study that examines this relationship, conducted in Tanzania, found “strong and sustained positive effects on food security among the participating households … in terms of access to food, food consumption, and quality of diet.”^32^^(p853)^ In Malawi, FFS programs were incorporated into a complex program designed to impact health and HIV vulnerability, and the intervention group had decreased odds of food insecurity.^33^ Similarly, the Jenga Jamaa II FFS group, when compared to the control group, had significantly increased HDDS and decreased HFIAS scores, thus reflecting improvements in household food access. It is important to note, however, that nearly half of all Jenga Jamaa II participants overall were still considered severely food-insecure at endline.^19^

Our study found that improvements in household food security did not appear to translate into improved nutritional status among children. This finding is aligned with several other reviews that failed to find consistent positive effects on nutrition from a variety of agricultural projects.^7^^–^^12^ Similarly, a systematic review was unable to find evidence of the effect of FFS programs on farmer health outcomes.^31^ While agricultural programs have sometimes been found to improve indicators associated with child nutrition and diet, significant changes in child anthropometry are rare. For example, one homestead agriculture and behavior change intervention in Burkina Faso demonstrated benefits in terms of reductions in child diarrhea and child anemia, with a marginal improvement in child wasting; no benefits were observed with respect to child underweight or stunting.^34^ In Nepal, a homestead food production and nutrition education program demonstrated similar findings, with improvements in maternal underweight and child anemia, but no significant gains in child anthropometric outcomes.^35^ Another study, in Cambodia, compared food security and nutrition outcomes between comparison areas receiving only agricultural programming (including an FFS) and intervention areas receiving both agricultural programming and nutrition education. While child dietary diversity improved significantly more in the intervention areas, child anthropometry *z* scores did not appear to be influenced by the intervention.^36^ There are many pathways through which agriculture can influence health, but successful changes in nutritional status are most likely when the emphasis is not on food production alone, but also on improving livelihoods, empowerment, and capacity.^37^

Improvements in household food security from 2012 to 2016 did not translate to improvements in child nutritional status.

Changes in nutritional status are most likely when the emphasis is not on food production alone, but also on improving livelihoods, empowerment, and capacity.

The concept of nutrition-sensitive agriculture has been promoted in recent years as a programming strategy that can raise incomes, increase female empowerment, bolster food production, and improve health. To accomplish these goals, however, the programs must explicitly set out to change child nutritional status. Furthermore, nutrition-sensitive agriculture seeks to connect disciplines and interventions on multiple levels,^38^ with programming that encourages education, behavior change, sustainability, and cross-sectoral collaboration.^39^ In this vein, the parent Jenga Jamaa II project included women's empowerment, FFS programs, and behavior change education components (the latter being focused on child health and accompanied by supplementary rations); households in the study reported here, however, benefited from the FFS programs only. In the future, increased nutritional impact may be found where beneficiaries are exposed to interventions that cut across multiple sectors.

In the case of our FFS intervention group, there are many potential reasons, apart from the FFS not being an integrated approach, for the failure of improved agricultural productivity and household food security to lead to improved child nutrition. Since child health outcomes are the product of multilevel factors at multiple life stages, improvements in household food security in this context may not outweigh the chronic impacts of poor sanitation, unstable livelihoods, and poverty.

For example, in rural DRC, access to improved sanitation and water sources is limited; diarrheal diseases are common among children (the 2013–2014 Demographic and Health Survey showed that almost one-fifth of children in the DRC had experienced diarrhea in the 2 weeks preceding the survey).^6^ There are physiological, social, and economic pathways through which long-term exposure to enteric infectious agents, and generally poor water, sanitation, and hygiene conditions, can increase the risk for chronic malnutrition.^40^ Thus, even if children are eating more and better food, their growth may still be compromised by, for example, poor absorption of nutrients and persistent immune response caused by subclinical infections and environmental enteric dysfunction.^41^

Furthermore, rain-fed agriculture is a risky undertaking, with harvests dependent on factors completely outside the control of the farmer. In the case of the Jenga Jamaa II FFS participants, crop diseases also posed a significant challenge, threatening harvests and perhaps attenuating some of the benefits of FFS participation, particularly among cassava-growing households. Finally, extreme poverty (in 2012, 65% of DRC's rural population lived below national poverty lines^42^) and low agricultural productivity are linked in a self-perpetuating cycle that exacerbates food insecurity in the DRC.^43^ Thus, optimal child growth is undermined on multiple levels by multisectoral challenges.

Children in the households participating in our FFS and control groups had baseline mean height-for-age *z* scores of −1.8 and −1.5, respectively, reflecting their poor nutritional status before the FFS programs commenced. It is difficult to reverse stunting that occurs before the age of 2 years.^44^ Although all children were under age 2 when enrolled in this study, some children were exposed to the program for only a short time before completing their first 1,000 days. As stunting is an indicator of chronic undernutrition, it is not likely that short-term exposure to an intervention will dramatically change the nutritional status of older children who already have poor nutritional status. More intensive and long-term programming may be needed to achieve sustained food security and nutrition improvements in low-resource and post-emergency contexts.

Stunting is an indicator of chronic undernutrition, and short-term exposure to an intervention is unlikely to dramatically change the nutritional status of older children.

Behavioral and knowledge barriers may also prevent improvements in household food security from affecting the nutritional status of children within those households. For example, focus group discussions with other Jenga Jamaa II participants in the DRC revealed that high-quality animal-source foods were frequently saved for the adult men in the households. Furthermore, women who worked in agricultural fields sometimes needed to leave their children with someone outside the household, and that person may not have been able to provide a diverse diet. In addition, caregivers were often unaware of ways to add nutritious foods to enrich the starchy porridges that are a common complementary food in the DRC.^45^

One limitation of the Jenga Jamaa II FFS intervention was lack of availability and timeliness of inputs. Seeds were sometimes delivered late and could not be planted at the optimal time in the growing season, thus limiting their usefulness.^46^ Insecurity also complicated both program delivery and data collection in some communities. The FFS intervention required a community-matched design; a randomized controlled trial would have been preferred but was not feasible. Selection bias may have resulted; program staff endeavored to enumerate comparable groups to minimize any impact, and key confounding variables were controlled for in the analyses through propensity score matching. Because the design was not randomized, however, we cannot rule out the possibility that factors other than the intervention were responsible for the changes observed. It is also possible that spillover from the intervention areas affected the control areas.

In addition, we made several decisions to streamline overall data interpretation: The food security results presented here include information only for participants who responded at both endline and baseline; interim measures were not included in the analysis. Due to lower child participation rates toward the end of the study, a multiple imputation approach was used for child nutrition outcomes: missing child outcomes were replaced by imputed values sampled from the fit of a linear regression model for the child outcomes at a given follow-up as a function of previous outcomes, as well as of child age and sex, then averaged using Rubin's method to obtain final estimates. It was not desirable to compare endline child nutrition outcomes to baseline because study enrollment occurred while many women were pregnant, making baseline anthropometric data unavailable for a large number of children.

Finally, data quality was a limitation for some indicators. We intended to measure agricultural yields, with the aim of reporting outcomes in terms of changes in agricultural techniques, crop production, household food security, and child nutritional status. However, due to concerns about the quality and consistency of agricultural yield data and our inability to validate the data collection approach, we concluded that the yield data were unreliable and should not be presented. We were thus unable to characterize FSS outcomes across the full pathway of outcomes as initially intended.

## CONCLUSIONS

Participation in the Jenga Jamaa II project's FFS intervention in South Kivu was associated with improvements in agricultural production and household food security, but it did not have a significant impact on child nutrition outcomes. To date, few studies have investigated the links between FFS programs, household food security, and child nutrition outcomes; this study therefore begins to address a gap in the evidence.

Participation in Jenga Jamaa II project's farmer field schools was associated with improvements in agricultural production and household food security, but did not have a significant impact on child nutrition outcomes.

Several recommendations emerge from the Jenga Jamaa II FFS experience that can inform future implementation of similar agricultural interventions. First, efforts to procure and ensure supply chain function should be established early so that input delivery and planting can happen at ideal times to maximize crop yields. In places where crop diseases are a major challenge, agricultural inputs should include resistant seeds and FFS curricula should include a strong focus on techniques to mitigate and prevent prevalent crop diseases. Since improved agricultural production will be less meaningful if farmers are unable to effectively sell their harvests, FFS programs and similar interventions should be paired with marketing training and, when appropriate, the formation of agricultural collection centers.

Further research is needed to understand whether FFS programs combined with nutrition-sensitive strategies and behavior change communication can improve child nutrition. Agricultural interventions similar to FFS programs may show increased impact on child growth outcomes if beneficiaries are exposed to focused and integrated programming specifically designed to improve nutritional status.

## Notes

**Peer Reviewed**

**Funding:** The Jenga Jamaa II project and this research were funded through a USAID Office of Food for Peace Cooperative Agreement (AID-FFP-A-11-00006).
